# Effect of add‐on melatonin on seizure outcomes and quality of sleep in epilepsy with idiopathic generalized tonic‐clonic seizures alone in adult patients: Cross‐sectional, randomized, double‐blind, placebo‐controlled clinical trial

**DOI:** 10.1002/brb3.2860

**Published:** 2023-01-01

**Authors:** Mehdi Maghbooli, Somayeh Alyan NajafAbadi, Ghazal MalekMahmoudi, Mohammad Hadi Molseghi

**Affiliations:** ^1^ Department of Neurology, Vali‐e‐Asr Hospital, School of Medicine Zanjan University of Medical Sciences Zanjan Iran; ^2^ Ayatollah Mousavi Hospital, School of Medicine Zanjan University of Medical Sciences Zanjan Iran

**Keywords:** anti‐seizure medication, generalized epilepsy, melatonin

## Abstract

**Background:**

Effective treatment of epilepsy is a major challenge in the field of neurology. Studies have suggested that melatonin can work in epilepsy with a good safety profile.

**Objectives:**

This study was performed to determine the effectiveness of melatonin in seizure outcomes, as well as the quality of sleep in patients with generalized epilepsy.

**Methods:**

In this cross‐over clinical trial study, 60 patients with epilepsy with idiopathic generalized tonic‐clonic seizures alone (EGTCS) and under valproic acid treatment received either melatonin or placebo with a washout period of 2 weeks intermittently. Outcome variables included a reduction in the severity and frequency of epilepsy besides improvement in electroencephalogram (EEG) abnormalities and sleep quality.

**Results:**

By adding melatonin, a decrease in the mean severity score of epilepsy (according to the Chalfont questionnaire) was 32.33 ± 9.24, while it was 5.58 ± 14.28 in treatment with placebo (*p* = .002). Evaluation of the number of attacks and EEG results did not disclose any therapeutic efficacy in treatment with melatonin versus placebo. The quality of sleep improved in 40% (first round) and 53.4% (second round) of subjects who received melatonin (*p* < .001).

**Conclusions:**

Considering that the addition of melatonin to routine anti‐seizure treatment was effective in reducing the severity of epilepsy and improving sleep quality, it seems that melatonin can be useful as an adjunct therapy for EGTCS in well‐defined circumstances.

## Graphical abstract

Recent data disclose that melatonin prevents seizures in animal models and could be effective and safe for decreasing epileptic manifestations in humans. This study indicated that the addition of melatonin to a routine antiepileptic regimen was effective in reducing the severity of epilepsy; it seems that melatonin can be used as an adjunct therapy for idiopathic generalized tonic‐clonic epilepsy in well‐defined circumstances.

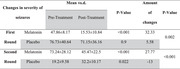

## 1 INTRODUCTION

Epilepsy is a prevalent neurological illness that affects approximately 50 million individuals worldwide, with a frequency of 0.5%–1% (Fisher et al., 2005). Because of poor health care and a bigger share of the pediatric population in poorer nations, it will rise to 7.4% (Goldberg‐Stern et al., 2012; Gupta, Gupta, et al., 2004). The primary epilepsy treatment is seizure control with a well‐tolerated anti‐seizure medicine (ASM) regimen. However, approximately one‐third of the 50 million people who suffer from epilepsy were treated with accessible ASMs, but some still experience recurrences due to a lack of beneficial impact (Brodie & Kwan, 2002; Dalic & Cook, 2016; González et al., 2015). These groups of patients need more aggressive treatment since monotherapy fails to control their attacks. Nevertheless, polytherapy often results in several unwanted side effects and comorbidities, such as neuropsychiatric concerns, mood disorders, behavioral issues, attention deficits, psychosis or cognitive impairments, and sleep disturbance. Furthermore, 20%−30% of patients experience adverse effects from ASMs, resulting in inadequate seizure control (Cramer et al., 2010). Unlike focal epilepsies, which have a variety of ASMs to choose from, idiopathic generalized epilepsy has a restricted number of therapeutic choices. Valproic acid is a well‐established first‐line therapy for focal and generalized seizures, although it is formally contraindicated in girls and women of reproductive potential who have failed to respond to previous therapies. Unfortunately, the requirement to avoid valproic acid makes managing idiopathic generalized epilepsy even more difficult (Tanoshima et al., 2015; Veroniki et al., 2017; Weston et al., 2016).

There was a diurnal divergence in the prevalence of epilepsy in human and animal research, suggesting the function of a time‐dependent biologic signal, and it appears that melatonin oscillations can impact this fact (Novakova et al., 2017; Reiter, 1986). Melatonin secretion in epilepsy has a variety of outcomes, including greater nighttime melatonin concentrations, a rise in melatonin levels during seizure events, and the absence of the typical daily secretion cycle melatonin implicated in seizure and sleep processes (Goldberg‐Stern et al., 2012; Paprocka et al., 2016; Rocha et al., 2018; Tarcin et al., 2020). Melatonin has been shown to suppress seizure activity, and melatonin‐treated animals with removed pineal glands experience fewer seizures than animals with pineal glands and not given melatonin. Melatonin appears to reduce seizure activity in human investigations of temporal‐lobe epilepsy (Albertson et al., 1981; Borowicz et al., 1999; Champney et al., 1996; de Lima et al., 2005; Mevissen & Ebert, 1998; Pévet, 2022; Rosenstein et al., 1990). Melatonin does not appear to have an impact on the normal electroencephalogram (EEG) (Paprocka et al., 2018).

Melatonin's anticonvulsant action has been linked to some different pathways. Its antioxidant, antiexcitotoxic, and free radical scavenging activities in the central nervous system (CNS) provide neuroprotection (Goldberg‐Stern et al., 2012; Gupta, Gupta, et al., 2004; Gupta, Aneja, et al., 2004; Gupta et al., 2005; Paprocka et al., 2018).

In generalized tonic‐clonic epilepsy, there have been no randomized controlled trials to test the benefit of melatonin adjunct treatment. Considering the failure of monotherapy, unwanted side effects of polytherapy, and restricted number of therapeutic choices in idiopathic generalized epilepsy, the goal of this study was to determine the effectiveness of melatonin in epilepsy with idiopathic generalized tonic‐clonic seizure alone (EGTCS) treatment (Khan et al., 2021).

## 2 STUDY DESIGN

This study was a randomized, double‐blind, placebo‐controlled clinical trial that recruited patients from Vali‐e‐Asr Hospital (Zanjan, Iran). Before patient enrollment, the study was approved by the Ethics Committee of Zanjan University of Medical Sciences (code: A‐11‐205‐18; https://ethics.research.ac.ir) and registered on the Iranian Registry of Clinical Trials website (code: IRCT20101209005352N4; https://irct.ir). This study was performed according to the Declaration of Helsinki and the International Council for Harmonization Good Clinical Practice guidelines.

## 3 PARTICIPANTS

Inclusion criteria were adult patients (aged between 18 and 60 years old) with confirmed EGTCS, a history of recent episodes (1–4 episodes) of seizure attacks (received valproic acid [10 mg/kg/day] for the last 6 months), and serum concentrations of 75–125 μg/ml at the time of enrollment. Because of the potential for drug interactions between ASMs and melatonin, to reduce the effects of drug interactions on the results of our study, the authors decided to use only valproic acid‐treated patients. Some drug interactions have been reported between melatonin and valproic acid; thus, for the evaluation of these interactions, during periodic examinations, patients were evaluated for short‐lasting feelings of depression, mild tremors, mild anxiety, abdominal cramps, irritability, reduced alertness, confusion, or disorientation.

Exclusion criteria were any recent traumatic brain injury, cerebral ischemia/TIA/stroke, neuroendocrine tumors, invasive neurosurgical/non‐invasive neuropsychiatric operation, pregnant women, a history of valproic acid or melatonin allergies, drug/alcohol addiction, and hepatic dysfunction. Informed consent was obtained from all participants or their parents/legal guardians.

## 4 RANDOMIZATION AND MASKING

Patients were randomly assigned in a 1:1 ratio to either melatonin + valproic acid or placebo + valproic acid groups. A computer‐generated randomization system was used to create the balanced blocks of patient numbers for each of the two therapy groups. Every patient received a specific six‐digit code, which was the same as a bottle of the drug. Only a nurse was informed of the contents of the bottles and the related code, and she was not involved in any of the study's processes until the data were collected. To ensure blindness, both active medicines were over tables equally, and matched placebo pills were administered in the double‐blind study, resulting in the same quantity of oral tablets being eaten. Patients, investigators, clinical researchers, and sponsor employees who supplied drugs, assessed outcomes, and processed data were unaware of the allocation until all of the data needed for the trial were collected.

## 5 PROCEDURES

**FIGURE 1:**
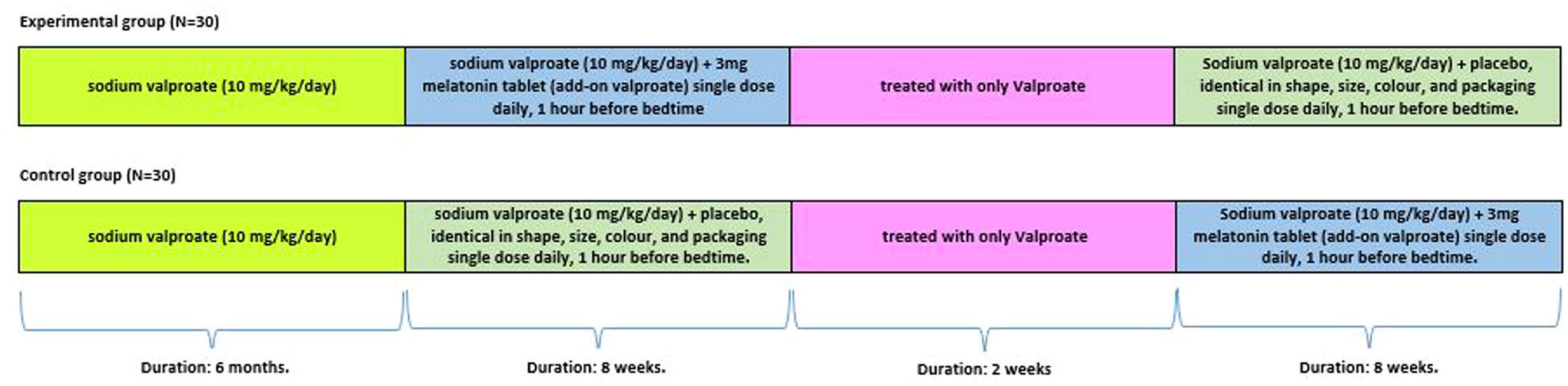
Diagram of study protocol

Following inclusion, blood samples (3 ml) were obtained from all patients, and the amount of valproic acid was determined using the high‐performance liquid chromatography method. In the experimental group, patients were given a 3‐mg melatonin tablet single dose daily (add‐on valproic acid) 1 h before bedtime and continued for 8 weeks (Verma et al., 2021). The dose of melatonin and the period of treatment were chosen according to the study by Gooneratne et al. (2012), who studied the pharmacokinetics of two different doses of melatonin. In the control group, patients were given a placebo, identical in shape, size, color, and packaging. The placebo tablets contained dicalcium phosphate along with other similar excipients. Afterward, patients were treated with only valproic acid for 2 weeks, and during this washout period, they did not receive any additional medication (including melatonin or placebo). Then, patients of both groups were exchanged (crossed‐over), and all the procedures were repeated (patients who received melatonin before were now given for placebo and vice versa). The study design is shown in Figure 1.

## 6 OUTCOMES

The outcomes of the study were assessed after 8 and 18 weeks of treatment and compared to the baseline. (1) The frequency and severity of seizures were measured according to the Chalfont Seizure Severity Scale (Duncan & Sander, 1991). (2) Sleep quality assessment was measured according to the Pittsburg questionnaire. (3) EEG changes were assessed for all patients with the same instrument (Nihon Kohden EEG‐1200) for 30 min, and all were interpreted by the same neurologist. Because of the lack of well‐defined grading of EEG abnormal findings, we used the below criteria to interpret the results:

1. Background rhythm: (a) normal or abnormal and (b) symmetric or asymmetric.
2. Abnormal discharges: (a) epileptiform and (b) slow.
3. Distribution: (a) focal, (b) regional, and (c) generalized.

See Supporting Information for data about questionnaires and EEG interpretation.

## 7 STATISTICAL ANALYSIS

Data were analyzed using SPSS version 18 (SPSS Inc, Chicago, Ill, USA). Descriptive statistics were used to summarize continuous data, whereas frequency and percentage were used to characterize categorical variables. For all of the outcome variables, descriptive statistics were computed and represented as mean ± SD or median and range, as applicable. The effects of add‐on melatonin versus placebo were compared using the independent *t* test (for variables with normal distribution) and Mann–Whitney test (for variables without normal distribution). The chi‐square test was used to compare two groups of categorical variables (e.g., gender). *p*‐Values less than .05 were considered statistically significant.

## 8 RESULTS

The entrance requirements were satisfied by 60 patients in total. Thirty individuals were given melatonin, and the other 30 were given a placebo. The patients in the valproic acid + melatonin group and valproic acid + placebo group did not differ significantly regarding mean age (38.49 ± 9.1 vs. 36.83 ± 12.7 years) and sex (male: 63% vs. 70%). No adverse reaction was observed from the melatonin therapy. The baseline characteristics of patients are shown in Table 1.

**TABLE 1:** Demographic and clinical characteristics of the study population

			Melatonin	Placebo	*p*‐Value
Gender (male %)			63%	70%	>.05
Age of onset of seizures (mean ± SD)			13.32 ± 8.7	14.78 ± 10.4	>.05
types of seizures			All were GTCS	All were GTCS	–
EEG characteristics		Normal	61.6%	63.3%	>.05
MRI characteristics		Abnormal	38.4%	36.7%	
		Normal	100%	100%	>.05
Family history		Abnormal	None	None	
			3.8%	3.5%	>.05
Frequency of seizures	before	1–5 Per month	80.2%	79.9%	>.05
		>5 Per month	19.7%	20.1%	
	after	1–5 Per month	91.8%	89.3%	>.05
		>5 Per month	8.2%	10.7%	
Concomitant therapies			All on monotherapy	All on monotherapy	–
Adverse drug events			Patients with drug reaction to valproic acid were excluded	Patients with drug reaction to valproic acid were excluded	–
Presence of drug resistance			Because of monotherapy, it is not applicable	Because of monotherapy it is not applicable	–

Abbreviations: EEG, electroencephalogram; GTCS, generalized tonic‐clonic seizure; MRI, magnetic resonance imaging.

The assessment of the severity of seizures showed that melatonin significantly reduced the severity of seizures, while placebo did not change this trend. After a washout period, the severity of seizures slightly increased, and after exchanging the groups, again, melatonin significantly reduced the severity of seizures (Table 2). Also, changes (differences between pretreatment and posttreatment scores) in the severity of seizures were analyzed. Our results showed that the efficacy of melatonin was significant (Table 2).

**TABLE 2:** Assessment of the severity of seizures between the study groups before and after treatment (scoring based on Chalfont questionnaire scale)

		Mean ± SD			
Changes in severity of seizures		Pre‐treatment	Post‐treatment	*p*‐Value	Amount of changes	*p*‐Value
**First Round**	Melatonin	47.86 ± 8.17	15.53 ± 10.84	<.001	32.33	.002
	Placebo	76.73 ± 40.64	71.15 ± 36.16	.9	5.58	
**Second Round**	Melatonin	73.24 ± 28.12	45.47 ± 22.5	<.001	27.77	<.001
	Placebo	19.2 ± 9.58	32.2 ± 10.17	.022	–13	

**TABLE 3:** Assessment of seizure frequency between the study groups before and after treatment

		Mean ± SD		
Changes in the frequency of seizures		Pre‐treatment	Post‐treatment	*p*‐Value
**First round**	Melatonin	2.2 ± 1.2	1.73 ± 1.48	.06
	Placebo	1.93 ± 1.03	2 ± 1.19	.3
**Second round**	Melatonin	1.73 ± 1.48	1.27 ± 1.38	.07
	Placebo	2 ± 1.19	1.87 ± 1.35	.8

Evaluation of the seizure frequency among patients showed that neither melatonin nor placebo significantly reduced the seizure frequency in the first and second periods after treatment (Table 3).

Also, the results of EEG interpretation were compared among patients in both groups and showed that melatonin and placebo did not make a significant difference in the EEG status (*p* = .443 and *p* = .247; Table 4).

**TABLE 4:** Qualitative assessment of electroencephalogram (EEG) interpretation between the study groups

		Percent			
Qualitative assessment of EEG interpretation		No changes (%)	Get better (%)	Get worse (%)	*p*‐Value
**First round**	Melatonin	60	20	20	.443
	Placebo	80	13.3	6.7	
**Second round**	Melatonin	53.3	20	26.7	.247
	Placebo	80	13.3	6.7	

**FIGURE 2:**
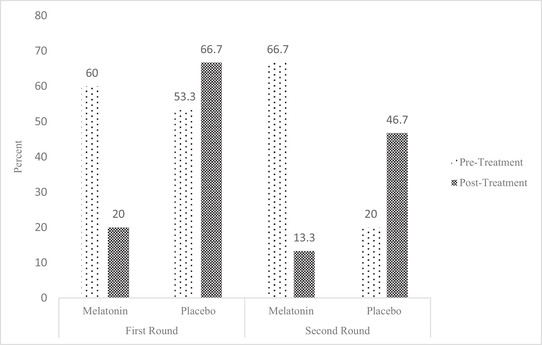
Assessment of the quality of sleep between the study groups before and after treatment

The assessment of the quality of sleep, according to the Pittsburg questionnaire, showed that melatonin significantly increased the quality of sleep, while placebo did not change this trend. After a washout period, again, melatonin significantly improved the quality of sleep (*p* < .001; Figure 2).

## 9 DISCUSSION

This study was performed on patients with EGTCS. According to our literature review, the majority of clinical investigations on the effect of melatonin in epilepsy have been performed on children, and fewer studies have been conducted on its influence on seizure outcome, neuroprotective effect, or sleep quality in adult epilepsy patients (Gupta, Gupta et al., 2004; Gupta, Aneja, et al., 2004; Gupta et al., 2005). The influence of melatonin on the severity of seizures, the number of epileptic seizures, the quality of sleep, and the pattern of the patient's EEG were investigated in this study. Thus, a cross‐sectional randomized, double‐blind placebo control clinical trial was conducted in which 30 patients with EGTCS were randomly treated with melatonin and 30 others with a placebo. For all patients, a washout period of 2 weeks was considered, and patients previously treated with melatonin cross‐treated with a placebo and vice versa.

Our findings suggested that patients treated with melatonin significantly benefited from reduced severity of epileptic seizures; however, in terms of the number of attacks, this reduction was not statistically significant. Sleep quality has also improved in patients treated with melatonin. There was no significant difference between the two groups in terms of EEG alterations. Melatonin is an endogenous hormone that inhibits brain excitability by acting on the MT1 and MT2 receptors (Liu et al., 2016). Melatonin has also been found to have anticonvulsant properties in animal models (LcV et al., 2002). Add‐on melatonin treatment has also been proven to improve clinical outcomes in several clinical trials (Gupta, Aneja, et al., 2004; Gupta et al., 2005).

Peled et al. (2001), in line with the results of our study, reported the antiepileptic effects of melatonin in their study population. However, their study population differed from ours; they evaluated the population of children with epilepsy who were resistant to treatment. In another study of patients aged 9–32 years, Goldberg et al. (2012) showed that melatonin could lead to a reduction in seizure length. This study could be interpreted in line with the results of our study. In our study, the severity of seizures in melatonin users decreased compared to the control group because seizure length was considered one of the criteria determining the severity.

Melatonin has a substantial influence on CNS in terms of regulating sleep and waking patterns (Banach et al., 2011). Melatonin regulates the electrical activity of neurons (Acufla‐Castroviejo et al., 1995) and enhances the inhibitory action of GABA neurotransmitters (Wan et al., 1999), both of which have suppressive effects on CNS (sedative, antiepileptic, analgesic, and hypnotic effects) and are produced by the pineal gland (Dawson & Encel, 1993; Golombek et al., 1996).

Melatonin has also been demonstrated to be safe in human tests at dosages ranging from 1 to 300 mg, with no negative side effects identified in the investigations (Doolen et al., 1998). Recently, in a double‐blind, placebo‐controlled clinical experiment, it has been shown that melatonin (10 mg) has no toxicological effects on participants (Guerrero & Reiter, 2002). In this study, we showed that melatonin could be useful in reducing the severity of epileptic seizures. Melatonin can be suggested as an adjunct drug in the treatment of EGTCS, but drug interactions between melatonin and other anti‐seizure drugs should be considered.

## AUTHOR CONTRIBUTIONS

*Manuscript writing, literature research, management of case, and final approval of manuscript*: Maghbooli and MalekMahmoudi. *Management of the case and editing the manuscript*: Alyan NajafAbadi and Molseghi. All authors have read and approved the manuscript.

## CONFLICT OF INTEREST

The authors declare no conflict of interest.

### PEER REVIEW

The peer review history for this article is available at https://publons.com/publon/10.1002/brb3.2860.

## Supporting information

Supplementary Information

## Data Availability

Data sharing is not applicable to this article as no datasets were generated or analyzed during the current study.
