# Supplementary material for: Effect of add‐on melatonin on seizure outcomes and quality of sleep in epilepsy with idiopathic generalized tonic‐clonic seizures alone in adult patients: Cross‐sectional, randomized, double‐blind, placebo‐controlled clinical trial
Source: Brain Behav. 2023 Jan 1;13(2):e2860. doi: 10.1002/brb3.2860 (PMC9927842; doi:10.1002/brb3.2860)
Supplement: Supplementary file 1 — Supplementary Information [file BRB3-13-e2860-s001.docx]

**Supplementary Information**

1. **Chalfont Questionnaire:** Seizure severity has been largely neglected in studies of patients with epilepsy and the evaluations of treatment. A seizure severity scale, which measures the components of seizures that cause patients the most disturbance, is presented with an assessment of the scale’s validity and reliability.
2. **Pittsburgh Questionnaire:** The Pittsburgh Sleep Quality Index (PSQI) is a self-rated questionnaire that assesses sleep quality and disturbances over a 1-month time interval. Nineteen individual items generate 7 “component” scores: subjective sleep quality, sleep latency, sleep duration, habitual sleep efficiency, sleep disturbances, use of sleep medication, and daytime dysfunction. The sum of scores for these 7 components yields 1 global score.
3. **Characteristics of EEG Measurement Equipment and Analysis:** This study was performed using NIHON KOHDEN EEG-1200 equipment according to the guidelines published by the American Clinical Neurophysiology Society (2006). The guidelines recommend the following items:

1. Record at least 16 channels.

2. Use the full 21-electrode array of the 10-20 system.

3. Both bipolar and referential montages should be used.

4. Electrode connections for each channel should be clearly indicated.

5. The pattern of electrode connections should be made as simple as possible, and the montages easily comprehended.

6. Bipolar electrode connections should run in a straight unbroken line with equal interelectrode distances.

7. The display of more anterior electrodes generally should be placed above those of more posterior locations.

8. At least some common montages should be used between studies for ease of comparison.

Electrode impedance was 100 ohm and usually no more than 5 kohm. Sometimes, impedance could not be kept within this window; thus, if the impedance had to be higher than 5 kohm, then the impedance should at least be approximately equal for the electrodes. The low-frequency filter (LFF) setting was 1 Hz, and the high-frequency filter (HFF) setting was 70 Hz. Amplifier sensitivity was initially set to 7 ìV/mm.

The most classic references of EEG indicated the adequacy of 30 min timing for a routine official EEG. All EEGs were done in the time interval between 8 AM and 12 midnight, while the administration of melatonin or placebo was at night, 60 min before sleep time.

A variety of background rhythms will be seen in EEGs, many of which are distinctly abnormal. We described background abnormalities based on the following 6 features: 1) symmetry,

2) continuity, 3) voltage, 4) organization (anterior-posterior gradient, predominant frequencies, and posterior-dominant rhythm), 5) reactivity (or variability), and 6) sleep architecture.

We used this principle: To determine if the EEG background is symmetric over both hemispheres, contrast their frequencies and amplitudes. First, use a longitudinal bipolar montage to compare the hemispheric frequencies and then use a referential montage to compare their amplitudes. The background may be:

1. Symmetric

2. Mildly asymmetric (if there is frequency asymmetry of less than 1 Hz or consistent amplitude asymmetry of less than 50%)

3. Markedly asymmetric (if there is frequency asymmetry of more than 1 Hz or consistent amplitude asymmetry of more than 50%).

There is physiological asymmetry. The nondominant hemisphere (commonly right) may have a slightly higher background amplitude compared to the dominant hemisphere (commonly left). Conversely, a higher dominant background or a markedly increased nondominant background is abnormal. Amplitude asymmetries were considered only significant for a difference of 50% or more.

**Abnormal Discharge Definition (Epileptiform and Slow):** Abnormal EEG activity includes ictal and epileptiform abnormalities and non-epileptiform abnormalities.

Epileptiform discharge includes spikes and sharp waves and combinations of these with slow waves. The EEG activity associated with generalized seizures comes in 4 common patterns, although some fewer common patterns also occur.

The common patterns are as follows:

• Three-per-second spike-wave

• Slow spike-wave (about 2.5/sec)

• Fast spike-wave (about 5/sec);

• Fast polyspike-wave (about 6-7/sec).

Both spikes and sharp waves are referred to as interictal epileptiform discharges (transients).

We used following definitions for abnormal Non-epileptiform EEG:

Discharges Associated with Epilepsy:

**Distribution Definition (Focal, Regional, or Generalized):** This is a matter of spatial distribution. Discharges can be described as focal, regional, lateralized, or generalized. Focal discharges are restricted to a few electrodes on one side. The term regional can be applied to a discharge that involves more than a few electrodes. If electrodes on one side are all affected, the discharge can be considered lateralized. Generalized discharges affect all electrodes on both sides. It is almost never the case that all electrodes are affected equally. Many generalized

Discharges have voltage predominance anteriorly, but there can be voltage predominance in a variety of regions. The terms diffuse and widespread are sometimes used synonymously with generalized but generally indicate a less clearly generalized field.
